# Sustained partial remission of a metastatic NEN using off-label immunotherapy with pembrolizumab

**DOI:** 10.18632/oncotarget.26906

**Published:** 2019-05-14

**Authors:** Anna Kathrin Stüven, Bertram Wiedenmann

**Affiliations:** ^1^ Department of Hepatology and Gastroenterology, Campus Virchow-Klinikum and Campus Charité Mitte, Charité-Universitätsmedizin Berlin, Berlin, Germany

**Keywords:** NET/NEC G2/3, pembrolizumab, immunotherapy, PD-L1

## Abstract

Neuroendocrine neoplasms (NEN) are a heterogeneous group of tumors, which can be histologically separated by primary location, proliferation rate and differentiation of tumor cells.

The therapeutic options and outcome depend on grading, staging and resectability of the tumor. Established treatment options of neuroendocrine tumors (NET) and carcinomas (NEC) are based especially on surgery, tumor specific medical treatments, peptide guided radioreceptor therapy (PRRT) and locoregional therapies.

We report about a patient diagnosed with a pancreatic, non-functional NET/NEC G2/3 with a proliferation rate of 20% at initial immunohistochemical diagnosis. During the course of the disease, the proliferation rate increased up to more than 50% over a period of 5 years. Due to loss of response to established therapies (i.e. systemic chemotherapy, targeted therapy and brachytherapy), an off-label immunotherapy with the PD-1 antibody pembrolizumab was initiated based on a 30% PD-L1 expression in tumor cells.

This report is the first demonstrating a partial remission of a pancreatic NEN using pembrolizumab monotherapy with a hepatic tumor volume reduction of at least 66%, combined with an improvement of the Karnofsky score rising from 60% to 100%. This case offers insight into the potential role of immunotherapy in a subgroup of neuroendocrine neoplasms.

## INTRODUCTION

Neuroendocrine neoplasms (NEN) are heterogeneous tumors, which can be differentiated between the broa d spectrum of low-proliferating and well-differentiated neuroendocrine tumors (NETs) to highly-proliferative and poorly differentiated, neuroendocrine carcinomas (NECs).

The current World Health Organization classification of gastrointestinal and pancreatic neuroendocrine neoplasms (GEP-NEN) uses the Ki 67 proliferation index to grade NETs as G1, G2, or NEN G3 with a high degree of proliferation [1, 2]. The group of NEN G3 is subdivided into NET and NEC, both characterized by significant differences in cell morphology and differentiation, proliferation index (Ki 67), progression-free survival (PFS) after chemotherapy, and outcome [3, 4].

Current guidelines for the treatment – as in the presented case - of a non-functional, metastatic pancreatic NET/NEC G2/3 recommend an anti-proliferative therapy with a somatostatin analogue [5] or systemic therapies including streptozotocin, temozolomide, everolimus or sunitinib [5].

Once NEN progress into an accelerated rate of proliferation and become a NEC, platinum-based chemotherapeutic regimens represent the treatment of choice [5, 6]. Given the almost obligatory, initial tumor response, therapeutic resistance also develops consistently. Therefore, dedifferentiated tumors/carcinomas have a poor prognosis and metastasize early to distant sites [6, 7].

In consequence, new alternative therapies are needed. Hence, immunotherapy has been explored as a potential and relevant therapeutic option for the treatment of NEC/T G2/G3. 

For immunotherapy, several receptor-ligand systems (RLS) influence immune responses. A well-established example of these RLS is programmed death 1 receptor (PD-1), a cell-surface molecule promoting self-tolerance by suppressing T-cell inflammatory activity. Immune checkpoint inhibitors offer the possibility to enhance the immune responses by intensifying the regulatory role of PD-1 [8–11]. One of the checkpoint inhibitors for PD-1 is the monoclonal antibody pembrolizumab, blocking the binding site of the receptor ligand PD-L1. The ligand is partially expressed by neuroendocrine tumors, especially in patients with metastatic GEP-NEC. PD-L1 expression of the tumor cells appears to be significantly associated with high-grade G2/G3 NEN [12] and is also significantly associated with a longer PFS in first-line treatment [8].

In addition, as recently reported, patients with Merkel-cell carcinoma expressing PD-L1 within the tumor tissue benefit from immunotherapy [13, 14]. It has been shown that, unlike chemotherapy, treatment with anti-PD-1 and anti-PD-L1 leads to a durable response in these patients [15]. As a result of this outcome, in September 2017 the European Commission approved avelumab, a PD-L1 inhibitor, to treat metastatic Merkel-cell carcinoma [16].

Based on the expression of PD-L1 and a sometimes-occurring high mutation rate, NEN G2/G3 are potential candidates for immunotherapy with for example pembrolizumab as checkpoint inhibitor [17–19].

This is the first case reporting a successful immunotherapeutic approach using pembrolizumab alone in a metastatic pancreatic neuroendocrine tumor.

## CASE REPORT

**Figure 1:**
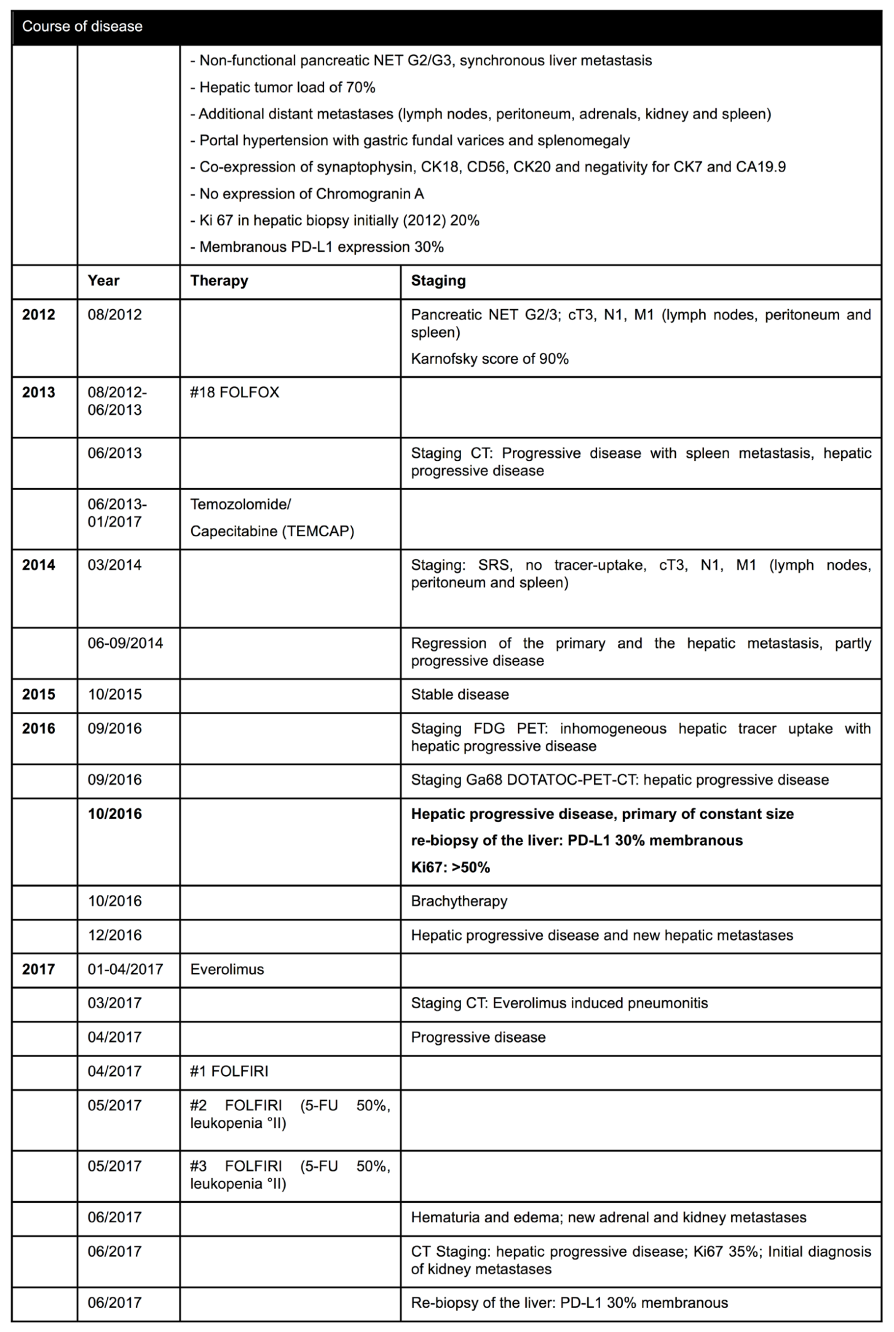
Course of disease As an indication of tumor wasting, tumor dissemination led to a weight loss of 10 kg over 8 months.

In this case study, a 55-year-old male patient was diagnosed in August 2012 with a non-functional pancreatic NET G2/G3 (according to the WHO 2010 classification) and synchronous liver metastasis with a hepatic tumor load of already 70%. In addition to the hepatic dissemination, metastasis also affected additional distant sites such as the spleen, kidneys, adrenals, peritoneum and lymph nodes. A table of the entire course of disease is provided in Figure 1.

**Figure 2:**
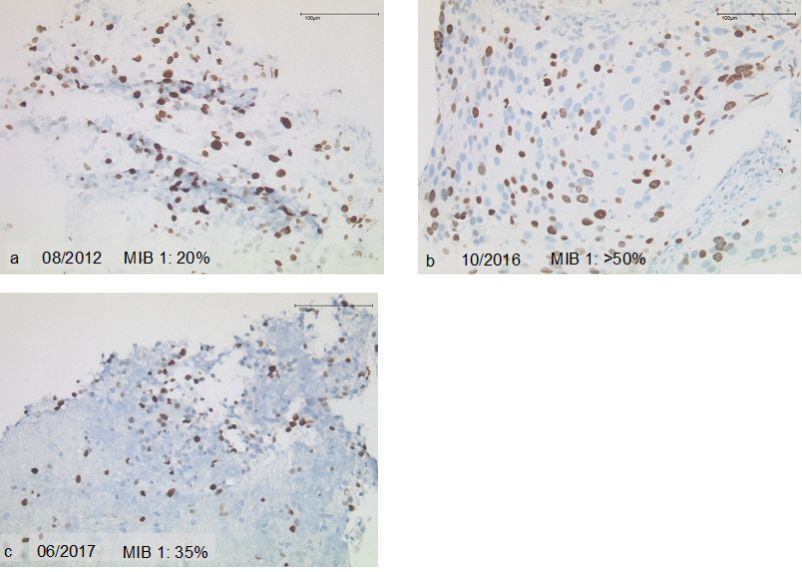
Proliferation during the course of disease **a.** IB 1 of 20% at initial diagnosis. **b.** MIB 1 of 50% after 4 years of treatment. **c.** MIB 1 of 35% at the beginning of treatment with pembrolizumab.

**Figure 3:**
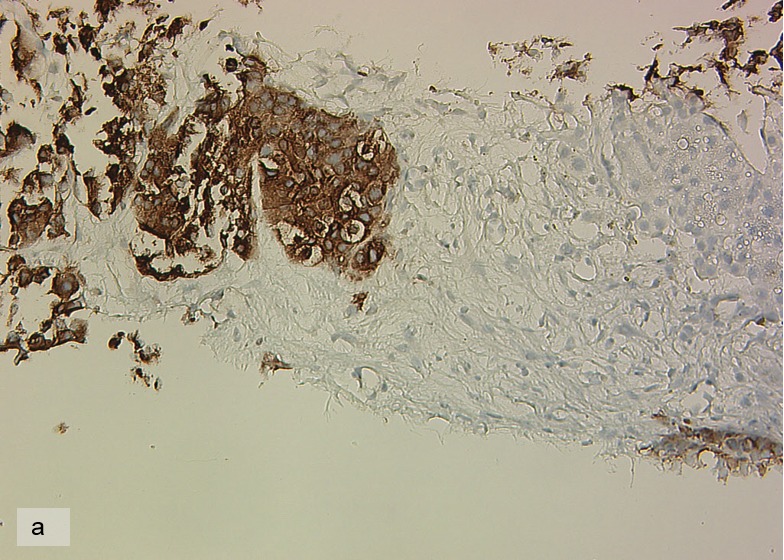
Synaptophysin-expression of the tumor cells.

Immunohistochemistry of a liver biopsy showed co-expression of synaptophysin, CK18, CK20, CD56 but negativity for Chromogranin A, CK7 and CA19.9. At initial diagnosis in 2012, Ki 67 was 20%, whereas in 2016 it had increased to >50% and in 2017 was reduced to 35%. Membranous PD-L1 expression was found in 30% of the tumor cells in the liver biopsy, both in 2016 and 2017. Figure 3.

*In vivo* somatostatin receptor imaging by Ga68-DOTATOC-PET-CT and tectreotide-scintigraphy showed a heterogenous somatostatin receptor expression with positivity for the primary tumor, but negativity for the liver metastases. Therefore, PRRT was excluded as a therapeutic option.

Due to the widespread dissemination of the tumor, curative surgery was not feasible. Consequently, first-line chemotherapy using FOLFOX was started in 2012 externally. Due to tumor progression, a second-line combination therapy using temozolomide and capecitabine was initiated.

Although the combination of temozolomide and capecitabine led to a stable disease for more than three years, eventually tumor resistance developed in 2016.

In October 2016, consecutive locoregional brachytherapy using an after-loading technique [20, 21] showed also fast progression of the hepatic metastases. Similarly, everolimus, an mTOR-inhibitor, was experimentally initiated despite a high Ki 67 > 50% [22, 23]. Everolimus had to be discontinued after three months based on pneumonitis as adverse effect. Another targeted drug, sunitinib, was excluded due to the expected lack of response to treatment and side effects such as arterial hypertension and bleeding based on the existing portal hypertension [24, 25].

Following all failed treatments, an additional large (78 mm in diameter) metastasis of the left kidney led to macrohematuria. The renal metastasis was treated with cyber knife and palliative local radiation, which led to a cessation of hematuria. 4th line systemic chemotherapy with FOLFIRI was initiated. Following also progression with FOLFIRI in June 2017, pembrolizumab, a highly selective, humanized monoclonal IgG4-kappa isotype antibody against PD-1 was started. Treatment began with 150 mg i.v. (2 mg/kg body weight) every 21 days and was deescalated to 100 mg every cycle due to pancytopenia [26]. For the following cycles, therapy with 140 mg was used without further side effects and recovery of hematopoiesis.

**Figure 4:**
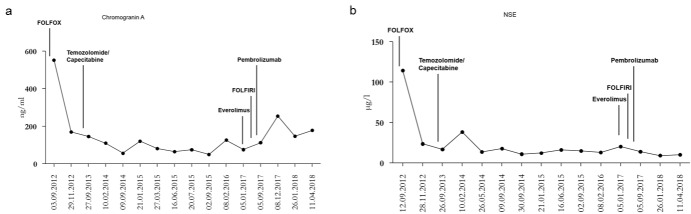
Tumor markers Chromogranin A and NSE during the different treatments. NSE seems to correlate with the effectiveness of the therapy, while Chromogranin A is not able to predict cytoreduction.

Until April 2018, monotherapy using PD-1-blocker led to a sustained partial remission with a hepatic tumor size reduction of at least 66% and a Karnofsky score of 100%. Figure 4.

**Figure 5:**
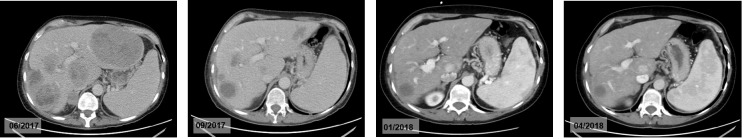
Metastases of the liver during the checkpoint-inhibition with pembrolizumab. It presents the hepatic tumor reduction of 66% from 06/2017 to 04/2018.

**Figure 6:**
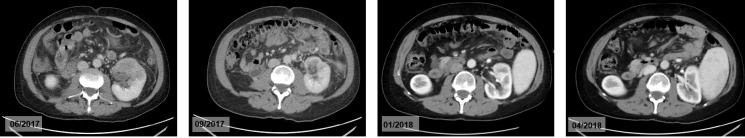
Renal metastases in CT-staging before and after the therapy with pembrolizumab. It reveals the distinct reduction of tumor size during a 10-month treatment.

Already three applications over a period of three months led to a partial remission with distinct regression of the hepatic, kidney and adrenal metastasis as shown by CT-imaging (Figure 5, 6). In addition, the general health condition including physical activity and health related quality of life (QoL) of the patient improved. Applying pembrolizumab, the patient gained 5 kg weight, stopped analgesics such as metamizole and tramadol, and resumed full time work again. Current physical examination after the thirteenth application of pembrolizumab over 9 months showed, that the liver gained normal size again, starting at initial diagnosis at mean corpuscular length of 190 mm in 08/2012 to 110 mm in 06/2017. In addition, CT-imaging revealed an impressive regression of the hepatic metastasis whereby in 11/2017 some lesions disappeared and other lesions as in segment 2/3 regressed from 60 x 40 mm in 09/2017 to 20 x 16 mm in 04/2018. Figure 5, 6.

Apart from a pembrolizumab induced pneumonitis of a low degree of severity with no clinical symptoms, we could not observe any other potential side effects known for pembrolizumab therapy such as fatigue, skin rush, diarrhea or allergic reaction during application [ 26].

Following 13 applications of pembrolizumab, therapy had to be paused due to pneumonitis and continued following a successful treatment with steroids. Until now, a persistent partial remission is observed. 

## DISCUSSION

To the best of our knowledge, the presented case represents the first report of successful treatment of a far advanced, metastatic pancreatic NET/NEC G2/G3 following partial, sustained remission over 13 months by now using a PD-1-blocker.

Until now, off-label treatment with pembrolizumab for pancreatic neuroendocrine tumors/carcinomas has not been successfully used. So far, only a case report on successful immunotherapy of a gastric NET/C exists (36). However, the presented case demonstrates quite well that off-label use may be considered in some cases of pancreatic NET/C once no other therapeutic option exists and PD-L1 expression in tumor cells is observed [27–29].

**Figure 7:**
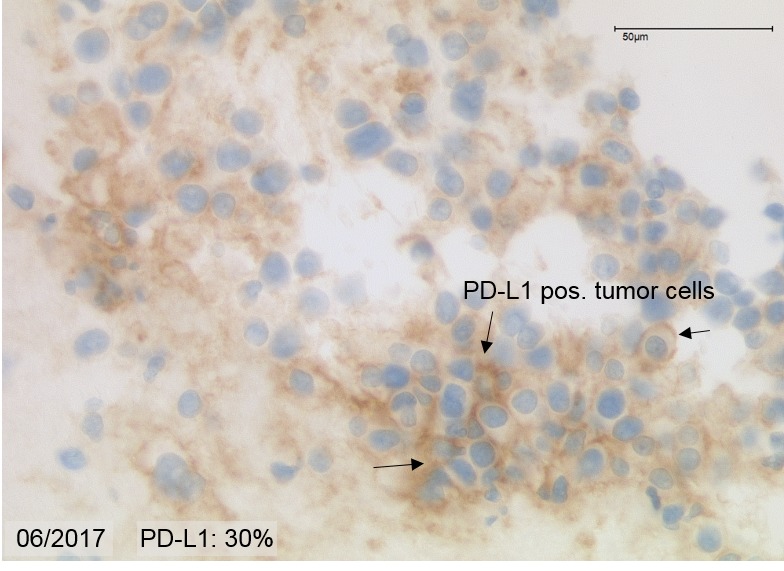
PD-L1 Immunoreactivity in 06/2017. In 30% of the tumor cells are immunoreactive.

The objective response of our patient is best explained by the high PD-L1-positivity, despite the high proliferation rate of Ki 67 of 35-50% [18]. In most reported cases so far, patients with high PD-L1 expression benefit from immunotherapy and often have a superior overall-response rate [30, 31]. Figure 7.

In consequence, PD-L1 expression appears to be a potential predictive marker. However, the validity is restricted by a broad diversity of assays with no standard antibody, and no standardized cut-off score for PD-L1 expression level, dividing tumor tissues into high- and low levels. 

In addition, latest research concerning modified immune response by exemplarily using vaccination, dendritic-cell-based therapy or indoleamine-2, 3-dioxygenase (IDO) inhibition in combination with PD-L1 related immunosuppressive mechanisms has to be considered in a future therapeutic prediction scenario [32–35]. For example, IDO inhibition combined with pembrolizumab therapy might enhance the odds ratio by improving T-cell activation in tumor cells [36]. Therefore, additional predictive biomarkers are necessary to evaluate patients that may benefit from immunotherapy. Aside from using PD-1 blockers, there is an educated guess, that the burden of neoantigens correlates with effectiveness of immunotherapies in NEN, like it is shown in other tumor entities [37]. It is well known, that GEP-NEC show a high chromosomal instability and microsatellite instability (MSI) rarely [38–41]. The presence of MSI seems to be associated with a better outcome, whereas the occurrence of mismatch repair antigens seems to be dependent on the microsatellite stable - NECs [18].

Furthermore, since we have already successfully treated another patient with pembrolizumab and ipilimumab long term [42], based on a high mutational burden, it is tempting to speculate that long-term remission may also be observed in the presented patient with elevated PD-L1 expression as a predictive marker for therapeutic response.

This would also in turn imply that control of typical side effects such as persistent pneumonitis has to be considered interfering with constant treatment. However, based on the observed durable response in Merkel-cell carcinoma, the experience at our site and the current data, it is reasonable to argue for a durable remission in the presented case [43, 44]. In case of future progressive disease, the combination of pembrolizumab with ipilimumab could be a therapeutic option for our patient. The combined therapy appears to lead to a longer PFS and a higher response rate than a monotherapy with ipilimumab. However, in comparison with a PD-1-antibody monotherapy, the occurrence of treatment-related adverse events of the combined therapy have to be considered very carefully and balanced against the therapeutic benefit [45].

Regarding patients with DNA-repair-deficiency, pembrolizumab also offers new treatment options in a neoadjuvant setting [46]. Increasing the extent of genetic instability by combined radiotherapy (RT) leading to an increased immunotherapeutic response has been described for rectal adenocarcinoma, melanoma, breast cancer and also NSCLC [47, 48]. It was also shown that RT is able to re-program the microenvironment of the tumor and is responsible for immune-mediated tumor rejection. For example, RT can upregulate the expression of PD-L1 [49, 50]. These therapeutic opportunities seem to be noteworthy for patients with neuroendocrine tumors as well.

Another interesting point for further work is to identify and evaluate the potential influences of pretreatment *versus* first-line treatment with pembrolizumab on the course of disease in neuroendocrine tumors. As shown for treatment with antibiotics, pretreatment might influence the composition of intestinal microbiota and therefore the outcome of patients treated with pembrolizumab [51, 52]. Current research shows that the gut microbiota seem to determine the effectiveness of anticancer immunotherapies and also provides new prognostic markers as a potential target for immunotherapy [53].

Further work might appreciate the transformation in proliferation during the period of treatment. As also observed in another case report of immunotherapy in neuroendocrine tumors, the proliferation index decreased during therapy [42]. This could be a working point for future evaluation of the tumor grading, leading to therapeutic adaptation or potential new treatment options.

In summary, the presented case report provides first evidence that immunotherapy may not only be effective in Merkel-cell and small cell carcinoma but also in the subset of NEN like pancreatic neuroendocrine tumors. Though the outcome cannot be generalized for any pancreatic NEN, in the presented case report monotherapy of pembrolizumab is able to reduce the tumor load and also leads to a dramatic gain of a health-related QoL. In this explicit case, a multimodal combination of systemic chemotherapy, local radiotherapy, and mTOR-inhibition seemed to be less effective for persistent remission and tumor-size reduction than the checkpoint inhibition of pembrolizumab. 

Therefore, the clinical course of the metastatic pancreatic neuroendocrine tumor G2/G3 with distinct partial remission in combination with an impressive gain of QoL and pain relief should arouse interest for the therapeutic capability of immunotherapy in neuroendocrine tumors.

## CONCLUSIONS

Metastatic pancreatic neuroendocrine tumors G2/3 can be successfully treated – in selected cases – with pembrolizumab, a highly selective PD-1-antibody, leading to a partial remission and thereby reducing the metastatic liver tumor load by at least 66% over at least one year. Furthermore, a high health-related quality of life and pain relief could be accomplished within several weeks after the initiation of immunotherapy.

So far, the molecular mechanisms are not entirely understood and explored; PD1-checkpoint inhibition appears to be a treatment option in a subgroup of metastatic neuroendocrine neoplasia. 

Multimodal treatments including immunotherapy can lead to a long survival of patients with metastatic NEN whereby sustained partial remission can be observed over a period of 6 years. Therefore, we suggest the use of immunotherapy as a therapeutic option for a subgroup of NEN-patients with distant metastases refractory to conventional treatments.
